# Monoclonal outbreak of Ralstonia solanacearum catheter-related bloodstream infection associated with contaminated package of normal saline solution in a tertiary care hospital

**DOI:** 10.3906/sag-2010-121

**Published:** 2021-06-28

**Authors:** Özlem GÜZEL TUNÇCAN, Murat DİZBAY, Hasan Selçuk ÖZGER, Sidre ERGANİŞ, Fatma Nur AKSAKAL, Meltem YALINAY, Gülendam BOZDAYI, Kayhan ÇAĞLAR

**Affiliations:** 1 Department of Infectious Diseases and Clinical Microbiology, Faculty of Medicine, Gazi University, Ankara Turkey; 2 Department of Medical Microbiology, Faculty of Medicine, Gazi University, Ankara Turkey; 3 Department of Public Health, Faculty of Medicine, Gazi University, Ankara Turkey

**Keywords:** *Ralstonia solanacearum*, outbreak, nosocomial, bloodstream infection, contaminated saline

## Abstract

**Background/aim:**

*Ralstonia solanacearum* is a very rare cause of infection in humans. There is no described nosocomial outbreak due to *R. solanacearum* so far. We determined *R. solanacearum* as the source of catheter-related bloodstream infection (CRBSI) outbreak.

**Materials and methods:**

This outbreak analysis was carried out in a 1000-bed tertiary care university hospital in Turkey. The outbreak analysis included hematology, oncology, nephrology, gastroenterology wards, emergency department, and intensive care units. The first case with *R. solanacearum* CRBSI was detected on May 20, 2019 and *R. solanacearum* was isolated in catheter blood cultures in 34 patients until October 3, 2019

**Results:**

Standard outbreak analysis procedures were applied. Culture samples were taken from the fluids administered via catheters. The cultures did not yield any bacteria. As a result of the investigation in storage area, it was found that there were leaks, air bubbles, and water drops inside the packaging of saline solutions. *R. solanacearum* was yielded in the cultures obtained from the surface of saline bags and the inner sides of plastic packings. To validate our hypothesis, a clonal analysis was performed using arbitrarily primed-PCR method and Sanger sequencing of the 16S rRNA gene for identification among isolates. All *R. solanacearum* isolates were monoclonal and identical.

**Conclusion:**

This is the first outbreak of *R. solanacearum* CRBSI described in a hospital setting. The source of the outbreak was a contamination in the surface of saline bags and the inner sides of plastic packings. Efficacy of an active surveillance system, accurate and rapid conduction of microbiological identification are essential for outbreak management.

## 1. Introduction

*Ralstonia* spp are emerging opportunistic pathogens within the nonfermenting gram-negative bacillus group that is present in both hospital and environmental settings and can be found in soil and water resources [1]. It is reported that *Ralstonia* spp. infections are increasing in parallel to the increase in the patient population at risk (older populations, neonates, and immunosuppressed and critically ill patients) and the implementation of more invasive procedures. *Ralstonia* spp. have been shown to be the causative agent of severe invasive infections, including bacteremia (especially central-venous-catheter-related), pneumonia, meningitis, osteomyelitis, etc. [1]. It can cause nosocomial outbreaks due to its resistance to disinfection procedures and its ability to live in water sources and low-nutrient conditions [2–4]. *Ralstonia pickettii* has caused contamination of pharmaceutical solutions in various countries, resulting in healthcare infections. *Ralstonia solanacearum* is a soilborne bacterium causing the widespread disease known as bacterial wilt and it is also the causal agent of Moko disease of banana and brown rot of potato. Although there are a few cases of infection, there has been no described outbreak due to *R. solanacearum* in humans [5]. 

In this article, we described a monoclonal outbreak of *R. solanacearum* catheter related bloodstream infection (CRBSI). The source of the outbreak was a contamination in the surface of saline bags and the inner side of plastic packaging. This is the first study that has proved *R. solanacearum* as the source of a nosocomial outbreak.

## 2. Materials and methods 

### 2.1.1. Hospital settings and outbreak

This outbreak analysis was carried out in a 1000-bed tertiary care university hospital in Turkey. The outbreak analysis included pediatric and adult hematology, oncology, nephrology, gastroenterology wards, emergency department, and intensive care units. These units, which include epidemic analysis, are located in different blocks and floors of the hospital and there is no staff mobility between them. The first case of *Ralstonia solanacearum* catheter related bloodstream infection (CRBSI) was detected on May 20, 2019 and *Ralstonia solanacearum* was isolated in catheter or/and peripheral blood cultures in 34 patients up until October 3, 2019.

### 2.1.2. Microbiological sampling 

After the literature review, case definition and hypothesis were conducted. All adults and pediatric patients with positive blood cultures were included in the study. Microbiological samples were obtained from environmental sources (distilled waters, saline, dextrose solutions, batticons, bedside oxygen jars, humidifiers, heparin solutions, tap water, and sinks) beside the blood cultures of patients.

## 2.2. Microbial identification 

Blood samples were evaluated using an automated blood culture system (Bact/ALERT® 3D, bioMerieux, Durham, NC, USA). Blood culture bottles signaling positive were removed from the Bact/ALERT® 3D system, aliquots were taken for subculture on the sheep blood agar and EMB agar media and then incubated at 37 °C for 24 h to 48 h. After the incubation period, identification and susceptibility testing was performed. Isolates grown on solid media were identified to the species level using the MALDI-TOF MS system (Bruker Biotyper; Bruker Daltonics, Bremen, Germany). The VITEK-2 antimicrobial susceptibility testing system (bioMerieux, Mercy L’Etoil, France) was used to determine the antibiotic susceptibilities of the isolates).

## 2.3. Molecular characterization of the isolates

We used the arbitrarily primed-polymerase chain reaction (AP-PCR) technique in order to study the clonal relationship among the *Ralstonia solanacearum* isolates. The M13 universal primers were used for this purpose. The AP-PCR was performed according to the method described by Prashanth and Badrinath [6]. The photographs of AP-PCR fingerprints were used for further analysis. To identify clonal strains, improved Sanger sequencing of the 16SrRNA gene technique was used as described by Chen et al. [7]. GenBank database [16S ribosomal RNA sequences (bacteria and archaea)] were used for nucleotide BLAST analysis. GenBank NCBI (2018). Sequence IDs [online].Website https://www.ncbi.nlm.nih.gov/nucleotide/CP022765.1?report=genbank&log$=nucltop&blast_rank=52&RID=4YN5B670016. [accessed 10 September 2018]DNA sequencing related to FASTA nucleotide reading were analyzed based on the phylogenetic similarly assay. The genomic results match with the MALDI-TOF proteomic profiles. The highest identity was selected as the identified species or genus [7].

## 3. Results

### 3.1. Epidemiological surveillance and investigation

*Ralstonia solanacearum* was first isolated from the blood culture [catheter] in a patient in the adult hematology unit on May 20, 2019. No new cases were detected in the same unit within next 2 weeks. However, blood cultures of seven patients yielded *R. solanacearum* in the 5th week. The infection control team investigated the epidemiological data, including predisposing risk factors of patients with *R. solanacearum* CRBSI. All of the patients (the range of age was between 1 month and 85 years old) were immunocompromised and had a long-term central venous or Hickman catheter. Insertion and maintenance care check-lists for central venous catheters and compliance with these check-lists were reviewed. Treatment solutions (saline, dextrose, or ringer lactate, etc.) and catheter care supplies were evaluated due to a previously reported possible relationship between *Ralstonia* spp.-associated bacteremia and contaminated solutions [2,3,8–10]. Standard outbreak analysis procedures were applied. Culture samples were taken from the fluids administered via catheters. The cultures did not yield any bacteria. As a result of the investigation in the storage area, it was found that there were leaks, air bubbles, and water drops inside the packaging of saline solutions. *R. solanacearum* was yielded in the cultures obtained from the surface of saline bags and the inner sides of plastic packings.

**Figure 1:**
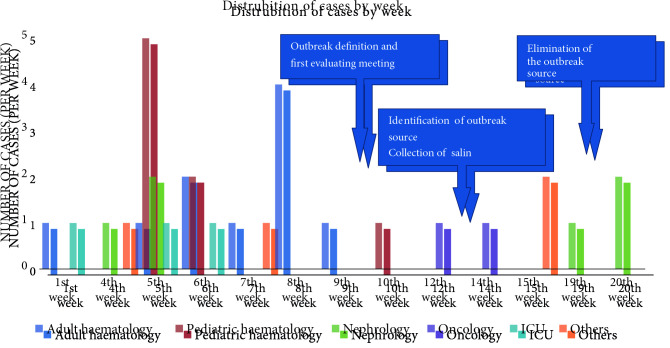
Distribution of cases by weeks.

Since the saline solutions were also used throughout the hospital, the surveillance of *Ralstonia* spp. was extended to the entire hospital. As a result of this surveillance, within the past 4 weeks *, R. solanacearum* catheter-related bacteremia was detected in the adult hematology unit (10 cases), pediatric hematology (8 cases), nephrology (6 cases), intensive care unit (4 cases), oncology unit (2 cases), and others (4 cases) (Figure 1).

It was thought that these contaminated packages caused the *R. solanacearum* catheter colonization or bacteremia especially in immunocompromised patients via hands of healthcare workers. 

**Figure 2:**
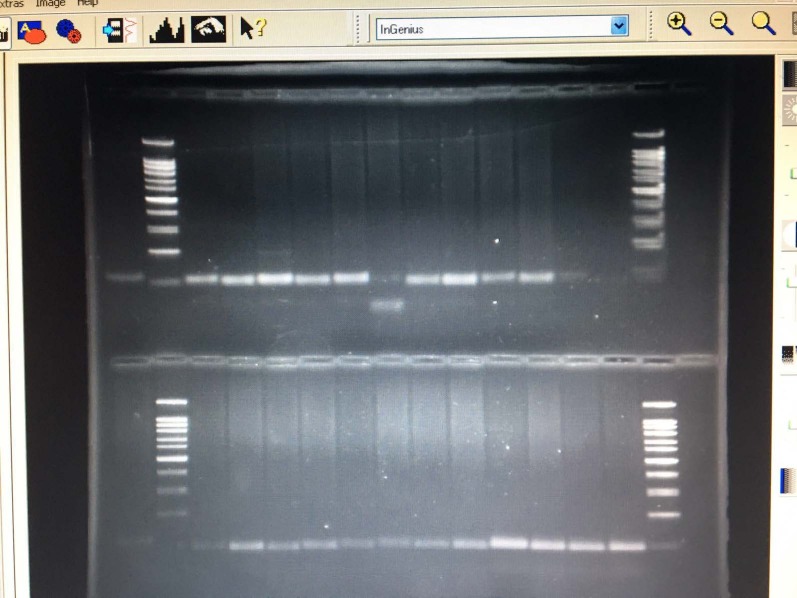
AP-PCR profiles of *Ralstonia solanacearum*.

All the saline solutions were withdrawn from the units and sent to the manufacturer. It was also reported to the health authorities. To validate our hypothesis, a clonal analysis was performed using the arbitrarily prime-PCR method and Sanger sequencing of the 16S rRNA gene for identification among *R. solanacearum* isolates. All *R. solanacearum* isolates were monoclonal and identical (Figure 2).

Antibiotic susceptibility test results revealed that all patients and environmental strains were susceptible to quinolones, penicillin, 3rd generation cephalosporins, carbapenems, trimethoprim-sulfamethoxazole, but resistant to aminoglycosides.

The outbreak, which has been proven to be associated with contaminated saline solution packs, was controlled by collecting all possible contaminated saline solutions distributed in units. No new cases were detected in the next 4 months period since the last case occurred. All patients were fully recovered through catheter removal and antibiotic treatment.

## 4. Discussion

*Ralstonia solanacearum* infections are not common in clinical settings, although a few cases have been reported with *R. pickettii* . In this outbreak analysis, a total of 34 cases of *R. solanacearum* -related bacteremia were reported. A monoclonal outbreak resulting from contaminated saline solution packs, which affected patients in different wards, was identified. This is the first described outbreak of *R. solanacearum* catheter-related bloodstream infection in hospital settings.

Although clinical infections with *Ralstonia* species are rare, disease progression to severity tends to be more serious once individuals are exposed. A large oncology hospital in Rome recently reported *R. mannitolilytica* infections among 12 oncology outpatients attending a day ward [11]. Liu et al. similarly reported three cases of bloodstream infections with *R. mannitolilytica* [12]. There is no report about the outbreak of *R. solanacearum* in humans until now. Shi et al. reported a case of hemophagocytic lymphohistiocytosis secondary to *Ralstonia solanacearum* infection [5]. *Ralstonia* spp. were considered unusual outbreak agents when compared with other outbreak agents. *Ralstonia* spp. with low virulence is considered a nonmajor nosocomial agent [3]. The development of an outbreak of bacteremia by this agent was made possible by direct access of the microorganism to the parenteral treatment area through contaminated saline packs.

The first nosocomial outbreak of *R. pickettii* , previously called *Pseudomonas pickettii* , was an outbreak of contaminated chlorhexidine-induced bacteremia in 1983 [13]. Since then, nosocomial outbreaks associated with *R. pickettii* ( *Pseudomonas pickettii* or *Burkholderia pickettii* ) have been reported in different years and in different patient groups [2,3,8,9,14]. When these outbreaks are evaluated, it is seen that contaminated solutions, drugs and disinfectants play an important role in *R. pickettii* -related outbreaks [3,10,15–17]. *R. pickettii* isolates can be found in hospitals and industrial production areas, especially in water resources, and can form a biofilm [4,18–20]. These literature data suggest that saline contamination, which may cause hospital outbreaks, may have developed during industrial production. In our outbreak, contamination was detected in saline solution packs, whereas intrinsic contamination of saline solution was not detected. This situation is thought to cause the number of cases to be limited.

In our outbreak, although the possible contaminated saline solution has been widely used throughout the hospital, the number of cases has been limited to certain units. This can be explained by the fact that bacteria with low virulence can only cause bacteremia in the susceptible host. Patients with hematologic malignancies, intensive care patients, patients with central venous catheters, transplant recipients, and newborns are reported to be susceptible to *Ralstonia* spp.-associated infections [1,2,21,22]. *R. pickettii* -related infections have also been reported in patients with diabetes, cystic fibrosis, chronic liver, and kidney disease [1,4,23–25]. There was an underlying hematologic malignant in approximately half of our cases. In these patients, malignancy or chemotherapy-related immune suppression was thought to facilitate the occurrence of *R. pickettii* -related bacteremia. Outbreaks of *Ralstonia* spp.-related bacteremia in patients with hematologic malignancies have been described in the literature [2,21,22]. Moreover, almost all of the patients we evaluated in the outbreak analysis had central venous or port catheters. Therefore, in addition to immune suppression and comorbid diseases, central venous catheters are also seen as an important risk factor for *Ralstonia* spp.-related bacteremia.

Ensuring outbreak control is possible with the continuation of effective surveillance, continuation of microbiological data analysis, and rapid and effective interventions. *R. solanacearum* -related bacteremia cases were detected at certain intervals within approximately 5 months. The epidemic was finally diagnosed 10 weeks after the first case due to clustering of cases in the adult hematology department. After the outbreak definition, source identification, identification of new cases, and planning of interventions to end the outbreak were carried out within approximately 1 month. However, the slowdown in elimination by collecting possible contaminated saline solutions spread throughout the hospital has led to the prolongation of the process between intervention and epidemic outcome.

It is stated that the MALDI-TOF method improves infection control applications by increasing the bacterial identification rate and makes a positive contribution to public health [26]. This contribution is valid in the outbreak analysis and control process. In our outbreaks, the use of the MALDI-TOF MS method for microbiological identification of *R. solanacearum* isolates facilitated and accelerated the identification of new cases and detection of possible sources.

A strength of our article is proving the identicality between clinical and environmental *Ralstonia* strains using molecular methods. In addition, using MALDI-TOF in identifying *Ralstonia* strains is another strength of our study. However, there are a few limitations to our study. For example, outbreak analysis was done only in wards which *Ralstonia* spp. were isolated from clinical samples. Due to retrospective aspects of the study, we could not perform further analysis in other wards. Therefore, the detected number of cases might be lower than the actual number of cases.

In conclusion, the number of immunosuppressed patients and the increase in invasive procedures increase the clinical effect and importance of low virulence pathogens such as *R. solanacearum.* This is the first outbreak of *R. solanacearum* CRBSI described in a hospital setting. The source of the outbreak was contamination in the surface of the saline bags and the inner sides of plastic packings. Efficacy of an active surveillance system and accurate and rapid conduction of microbiological identification are essential for outbreak management. Accurate identification of the outbreak agent facilitates the identification and control of possible outbreak sources with the evaluation of previous data.
